# Nanoparticle-Enhanced Engine Oils for Automotive Applications: Thermal Conductivity and Heat Capacity Improvements

**DOI:** 10.3390/molecules30132695

**Published:** 2025-06-22

**Authors:** G. D. C. P. Galpaya, M. D. K. M. Gunasena, D. K. A. Induranga, H. V. V. Priyadarshana, S. V. A. A. Indupama, E. R. J. M. D. D. P. Wijesekara, M. I. Ishra, M. M. M. G. P. G. Mantilaka, K. R. Koswattage

**Affiliations:** 1 Center for Nanodevice Fabrication and Characterization, Faculty of Technology, Sabaragamuwa University of Sri Lanka, Belihuloya 70140, Sri Lanka; chanakagalpaya@gmail.com (G.D.C.P.G.); kasundi@tech.sab.ac.lk (M.D.K.M.G.); ashaninduranga@tech.sab.ac.lk (D.K.A.I.); vimukkthi@tech.sab.ac.lk (H.V.V.P.); amalka@tech.sab.ac.lk (S.V.A.A.I.); dasith@tech.sab.ac.lk (E.R.J.M.D.D.P.W.); ishraiqbal@yahoo.com (M.I.I.); mantilaka@gmail.com (M.M.M.G.P.G.M.); 2 Department of Biosystems Technology, Faculty of Technology, Sabaragamuwa University of Sri Lanka, Belihuloya 70140, Sri Lanka; 3 Department of Engineering Technology, Faculty of Technology, Sabaragamuwa University of Sri Lanka, Belihuloya 70140, Sri Lanka

**Keywords:** nanofluids, engine oil, thermal conductivity, thermal diffusivity, specific heat capacity, fullerene C_60_, TiO_2_, Fe_2_O_3_, rGO

## Abstract

The poor thermal and physical properties of conventional engine oils limit vehicle performance and durability. This research aims to investigate the effect of nanoparticles such as fullerene C_60_, titanium dioxide (TiO_2_), iron oxide (Fe_2_O_3_), and reduced graphene oxide (rGO) nanoparticles on 10W30 Mobil engine oil. In this study, the effect of nanoparticle concentrations at different mass fractions (0.01, 0.05, and 0.1) was examined within the temperature range 30–120 °C. The nanofluids were prepared using a two-step direct mixing method and thermal properties were measured using a LAMBDA thermal conductivity meter, which uses the transient hot wire method according to the ISO standards. Due to the low concentrations of the nanofluids, surfactants were not required at all, and the stability of the nanofluids was visually monitored over a period of four weeks. Accordingly, the largest improvement in thermal conductivity occurred with TiO_2_/10W30 at a mass fraction of 0.1 wt.% at 80 °C, and the specific heat capacity improved due to Fe_2_O_3_/10W30 addition at a mass fraction of 0.1 at 70 °C; these were 5.8% and 14.4%, respectively, for the base oil. Thermal diffusivity remained largely unaffected by the addition of the nanoparticles, and fullerene C_60_ showed no significant effect on any thermal property. It was concluded that the thermal properties of the engine oil were considerably enhanced by the added nanoparticles at different weight fractions and temperature values.

## 1. Introduction

Technology has undergone considerable evolution in all the different sectors of industry in recent decades. As a result, the need to achieve better results through optimizing benefits, minimizing losses, and, above all, improving methods’ performance and discovering new solutions has led to a situation such that almost all research groups have discovered the benefit of nanotechnology in their respective fields of study. Insufficient lubrication in mechanical assemblies causes most mechanical part failures in the industry [1,2,3]. Lubricating oil is mainly used to reduce the wear and friction of mechanical parts. However, it has some other major properties such as thermal properties, which help to improve machines. There are several traditional coolants and lubricating oils, like water, oil, and ethylene glycol, which are used in industrial applications, but they do not meet the thermo-physical requirements due to the base fluids having their own limitations. These liquids have constant values for their properties, so the only way to improve their heat transfer features is to use a device, that is to say, through augmenting the heat exchange area or the flow rates of coolants. Nevertheless, this solution implies a higher heat exchange potential, but it does not enhance the efficiency of the procedure, which is a relevant concern. Therefore, researchers have looked for some potential solutions to enhance the properties of lubricating oil, and they tend to introduce nanotechnology. Nanotechnology is an emerging field, which plays an important role in the development of innovative technology to produce new products with improved performance that utilize less energy and reduce harm to the environment [4,5,6,7,8]. Nanoparticles, typically defined as particles with at least one dimension less than 100 nanometers (nm), have garnered significant attention across various fields due to their unique properties and wide-ranging applications. Their introduction into scientific discourse has sparked innovation in fields such as medicine, electronics, environmental science, and materials science. For instance, ancient Egypt’s renowned blue pigment, cuprorivaite, predominantly consists of quartz and nanoparticulate glass [9]. Furthermore, the properties of the nanoparticles mainly depend on their sizes, with smaller particles exhibiting more unique characteristics compared to their larger counterparts [10]. In material science, nanoparticles offer exciting opportunities for tailoring the properties of materials at the nanoscale. By controlling the size, shape, and composition of nanoparticles, researchers can engineer materials with novel characteristics, such as enhanced strength, conductivity, and catalytic activity. These materials find applications in fields as diverse as the automotive, aerospace, and construction sectors, driving advances in performance, durability, and functionality. However, alongside their vast potential, nanoparticles also pose challenges related to their synthesis, characterization, and environmental impact. Ensuring the safe and responsible use of nanoparticles requires rigorous investigation into their toxicity, biocompatibility, and long-term effects on human health and the environment. Due to the small size and shape of nanoparticles, they have unique properties. Some of these properties include the following: Nanoparticles have a very large surface-area-to-volume ratio, which means that they have a large surface area relative to their size. The quantum effect property can lead to changes in the electronic, optical, and magnetic properties of materials; nanoscale materials can exhibit this quantum effect, but larger materials do not. Furthermore, due to the high surface area of nanoparticles, they have higher reactivity, better optical properties, and more tunable properties than other larger-sized particles. There are three main types of nanomaterial, which are metal nanoparticles (e.g., gold, silver, platinum, and iron), metal oxide nanoparticles (titanium dioxide (TiO_2_), zinc oxide (ZnO), and iron oxide (Fe_2_O_3_)), and carbon-based nanomaterials (fullerenes, carbon nanotubes (CNTs), graphene, and carbon dots), and there are a few other types of nanomaterial, such as polymeric nanoparticles (PLGA, polyethylene glycol, and chitosan), quantum dots, ceramic nanoparticles (alumina (Al_2_O_3_), silica (SiO_2_), and titania (TiO_2_)), and lipid-based nanoparticles (liposomes, solid lipid nanoparticles, and nanostructured lipid carriers). The term nanofluid was initially defined in 1995 by Choi, who coined it when working on a research project at Argonne National Laboratory [11]. According to the previous studies, Choi defined it as an innovative new class of heat transfer fluids that can be prepared by suspending nanoparticles in conventional heat transfer fluids and are able to enhance the thermal conductivity and convective heat transfer performance of their base fluids. In this way, their values are orders of magnitude higher than those of traditional base fluids such as ethylene glycol, water, and oils [12,13,14]. Nanofluids’ behavior depends on a few important parameters such as particle size, concentration, shape, nanoparticle material, base fluid nature, the sonication time of samples, and the method employed by the manufacturer for the pH value of the dilution. There are several nanofluid preparation methods that are used by scientists. To optimize the thermo-physical properties of nanofluids, a stable nature/homogeneous suspension is essential, and that is obtained through the achievement of successful synthesis processes [15]. The two main methods are the two-step direct mixing method and the one-step method. As the name of the method suggests, the two-step method has two steps: the first step is when nanoparticles are prepared, and the second step consists of the dispersion of nanoparticles into a base fluid. The dispersion of nanoparticles into the selected base fluid requires simple techniques such as the addition of surfactants to the fluids, changing nanofluids’ pH value, stirring, and both mechanical and ultrasonic agitation to obtain stable samples, since the minimization of particle aggregation and dispersion improvement will be achieved. As described, in the two-step process, nanoparticle synthesis and nanoparticle dispersion into the base fluids are carried out in two different stages, but in the case of the one-step process, both steps are performed at the same time. An advantage of this method is that the process of the drying, storage, transportation, and dispersion of nanoparticles is avoided, so the agglomeration of nanoparticles is minimized and the stability of the fluids is maximized [16]. This process is highly costly when compared with the two-step process, and another matter regarding the chemical one-step process is that the residual reactants are left in the nanofluids due to incomplete reaction or stabilization. It is difficult to elucidate the nanoparticle effect without eliminating this impurity effect. So, considering the industrial and commercial scale, a two-step process is better than a one-step process. A measure of a fluid’s ability to conduct heat, with higher values indicating better heat transfer capabilities, is called thermal conductivity. When considering nanofluids in engine oil, thermal conductivity can be named as the most imperative parameter. Generally, the addition of nanoparticles to engine oil causes an enhancement in the thermal conductivity, due to the thermal conductivity of nanoparticles being higher than that of base fluids and the formation of a conductive network or pathways within the nanofluid. Generally, the thermal conductivity of engine oil is inversely proportional to the temperature due to the distance between two molecules increasing with temperature, which results in their mean free path increasing. This reduces the collision probability of molecules. In nanofluids, nanoparticles are placed in liquid molecules to reduce the distance and make a thermal bridge between two molecules, which enhances the heat-transferring process by passing the kinetic energy. Experimentation results have shown that nanofluid thermal conductivity is dependent on several key factors such as base fluid type [17,18,19,20,21], surfactants [22,23,24,25,26,27], concentration [28,29,30,31], temperature [19,20,28], and characteristics of nanoparticles, such as nanoparticle size [17,31] and nanoparticle shape [28,32]. Furthermore, thermal conductivity gives an idea about the ability of a substance to conduct heat, and its units are W/mk. Simply put, when high-thermal-conductivity particles are added to a fluid, the high value of thermal conductivity of the nanofluid can be expected. Furthermore, there are several mechanisms that significantly affect the thermal conductivity enhancement of nanofluids, and they are Brownian motion, aggregation, and the formation of an orderly liquid layer around nanoparticles. These theoretical models describe how nanoparticles’ behavior impacts the thermal conductivity of nanofluids. According to Sulgani et al., the thermal conductivity of Al_2_O_3_-Fe_2_O_3_ nanoparticles added to 10W40 engine oil-based nanofluid with five different mass fractions, 0.25, 0.5, 1, 2, and 4, improved even at the smallest value of the mass fraction, and the largest improvement occurred at a mass fraction % of 33% of the base oil [33]. Sathishkumar et al. performed an experimental investigation of multi-walled carbon nanotubes added to 20W50 engine oil-based nanofluids with two weight concentrations of 0.2 and 0.4. Their results proved that the thermal conductivity was enhanced by 6.3% and 10.5% at 0.2 and 0.4 wt.%, and this enhancement occurred due to the higher thermal conductivity of multi-walled carbon nanotubes with respect to the engine oil [34]. Sukkar et al. experimentally proved that in CuO and TiO_2_ added 15W40 engine oil-based nanofluids with different weight fractions, thermal conductivity was enhanced by 7.27% in CuO/oil and 4.54% in TiO_2_/oil at 0.1 [35]. Thermal diffusivity can be called the rate of heat transfer through a medium. There is not much recent research carried out by scientists regarding the thermal diffusivity of engine oil-based nanofluids. However, recent research work can be found about the thermal diffusivity of 10W30 engine oil-based fullerene-C_60_-, TiO_2_-, and Fe_2_O_3_-added nanofluids in the temperature range 30–120 °C at a mass fraction of 0.01, which was carried out by us in 2024. The experimental results proved that there was no considerable change in the thermal diffusivity of nanofluids in contrast to that of the base fluid [36]. Specific heat capacity depends on the type of material, which makes some materials better at storing heat. But when it comes to nanofluids, it depends on the type of material, base fluid, nanomaterial concentration, and temperature. Generally, the specific heat capacity of nanofluids is higher than that of the base fluid due to the nanoparticles. Liu et al. reported a drop in the specific heat capacity of Gr–water nanofluid from 3.915 J/g.K for pure water at 20 °C to 3.875, 3.834, and 3.768 J/g.K for 0.2, 0.4, and 0.8 wt.%, respectively, representing a drop of up to 3.75% [37]. Elsaid et al. studied graphene-based nanofluids’ thermo-physical properties and mentioned that the nanoparticles had lower specific heat capacity values when compared with all base fluids [38]. Stalin et al. experimentally investigated that GO- and Al_2_O_3_-added water-based nanofluid showed a maximum reduction in the specific heat capacity ratio of 7% at 20 °C at a mass fraction of 0.15 [39].

The novelty of this study was using two different types of nanoparticles which were carbon-based and metal oxide nanoparticles with Mobil 10W30 engine oil at three different weight ratios. Furthermore, all thermal properties were systematically measured in the temperature range 30–120 °C with a step size of 10. To the best of our knowledge, this is the first experimental study that evaluates the thermal conductivity, diffusivity, and specific heat capacity of Mobil 10W30 engine oil modified with a dual-nanoparticle approach in a broad temperature range, offering valuable insights into the design of next-generation nanolubricants.

## 2. Results and Discussion

Four different types of nanoparticles were used to prepare the nanofluids, which were based on 10W30 engine oil. Fullerene-C_60_, which is also known as buckminsterfullerene, has unique properties due to its structure, such as stability, electrical conductivity, optical properties, photochemical reactivity, biocompatibility, magnetic properties, etc. Figure 17a, which is an SEM image of fullerene-C_60_, proves that the average particle size is about 100–200 nm.

Anatase-type TiO_2_ nanoparticles have some unique properties such as thermal stability, mechanical properties, a high dielectric constant, semiconductor behavior, and optical properties due to their high surface area, chemical stability, and high surface reactivity. Figure 17b shows an SEM image of anatase-type TiO_2_, and it proves that the average particle size is about 30–60 nm.

The third type used for the experiment was iron oxide nanoparticles which have several unique properties such as magnetic properties due to their ferromagnetic behavior, a high surface area-to-volume ratio due to their small size, which enhances their activity and adsorption capacity, electrochemical properties due to their high theoretical specific capacity, chemical stability, and tunable properties. Figure 17c shows an SEM image of Fe_2_O_3,_ and it proves that the average particle size is about 40–70 nm. The morphology of the TiO_2_ and Fe_2_O_3_ nanoparticles was spherical to some extent, which provided a good rolling mediator inside the engine oil.

Reduced graphene oxide is the nanoparticle with the highest thermal conductivity (2000 W/mK) property and it has several unique properties such as electrical conductivity due to the restoration of the sp2 carbon network during the reduction process; high surface area, allowing for high adsorption capacity and increased reactivity; mechanical strength; flexibility, allowing easy dispersion in solutions; chemical stability; surface functionalization; etc. Figure 17d shows an SEM image of rGO, and it proves that the average particle size is about 100–200 nm, and the nanoparticles are sheet-like structures to some extent.

### 2.1. Thermal Conductivity

Thermal conductivity can be defined as the ability of a material to conduct heat, and nanofluids’ thermal conductivity depends on several factors such as nanoparticle size, nanoparticle type, concentration, base fluid, and temperature.

**Figure 1:**
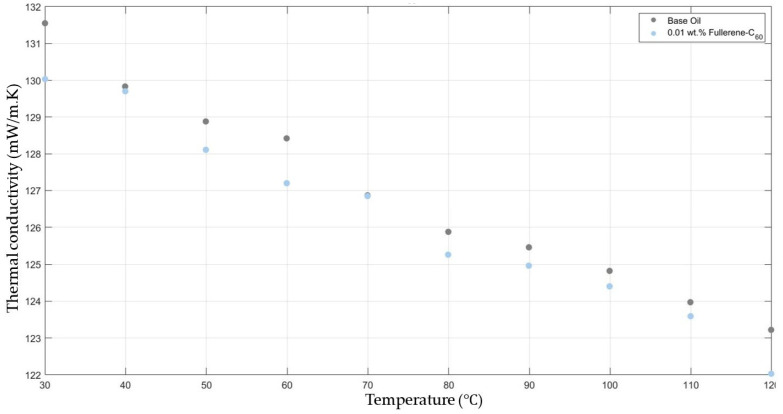
Effect of temperature on thermal conductivity of fullerene-C_60_ 0.01-added 10W30 engine oil-based nanofluid.

Figure 1 compares the thermal conductivity results of 10W30 engine oil with fullerene-C_60_ 0.01 added within the temperature range 30–120 °C. Both the base fluid and nanofluids showed a decrease in thermal conductivity when increasing the temperature, keeping the graphs’ gradients approximately equal. And there was no considerable enhancement in the fullerene-C_60_-added nanofluids’ thermal conductivity when compared with that of the base fluid.

**Figure 2:**
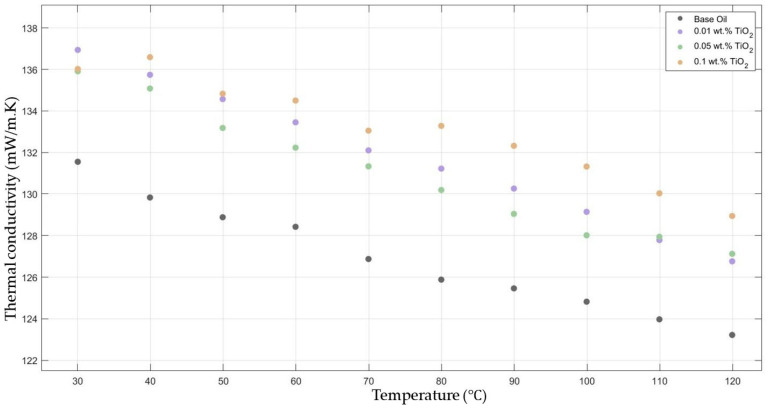
Effect of temperature on thermal conductivity of TiO_2_ 0.01-, 0.05-, and 0.1-added nanofluid with base fluid.

Figure 2 compares the thermal conductivity results of 10W30 engine oil with different weight ratios of TiO_2_ added within the temperature range of 30–120 °C. The graphs demonstrate significant enhancements in the nanofluids’ thermal conductivity compared to the base fluids’ thermal conductivity, while showing the highest enhancement with 0.1 TiO_2_-added nanofluid.

**Figure 3:**
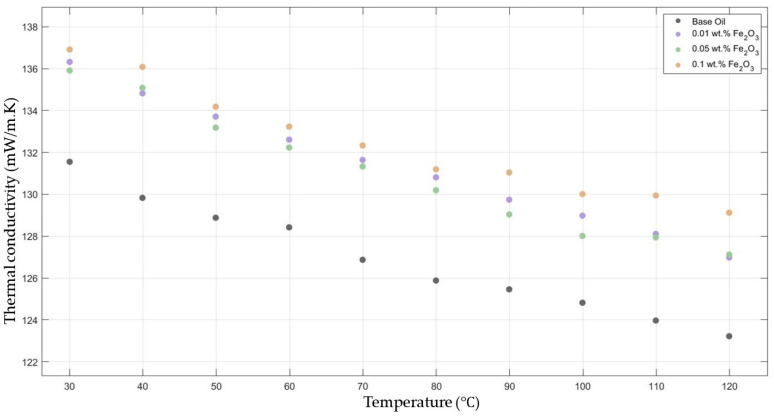
Effect of temperature on thermal conductivity of Fe_2_O_3_ 0.01-, 0.05-, and 0.1-added nanofluids with base fluid.

Figure 3 compares the thermal conductivity results of 10W30 engine oil with different weight ratios of Fe_2_O_3_ added within the temperature range 30–120 °C. The graph clearly proves that there was a significant enhancement in the thermal conductivity of Fe_2_O_3_-added nanofluids in contrast to that of the base fluid while maintaining the highest enhancement with the 0.1 Fe_2_O_3_-added nanofluid.

**Figure 4:**
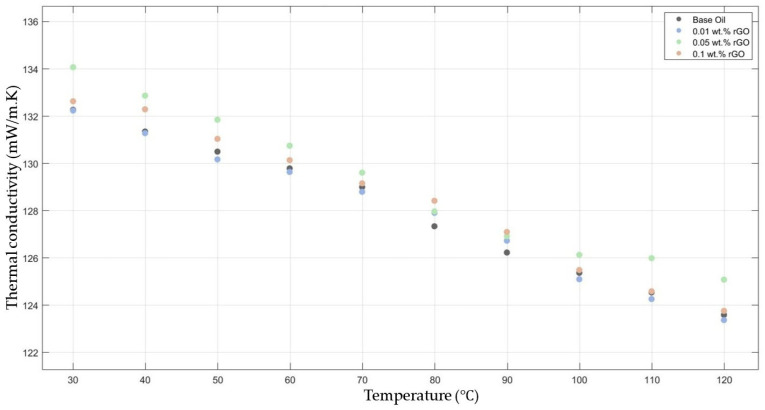
Effect of temperature on thermal conductivity of rGO 0.01-, 0.05-, and 0.1-added nanofluids with base fluid.

Figure 4 compares the thermal conductivity results of 10W30 engine oil with different weight ratios of rGO added within the temperature range of 30–120 °C. The graph demonstrates enhancements in the thermal conductivity of 0.05 and 0.1 rGO-added nanofluids; however, the 0.01 rGO-added nanofluid did not show much enhancement when compared with the base fluid thermal conductivity.

**Figure 5:**
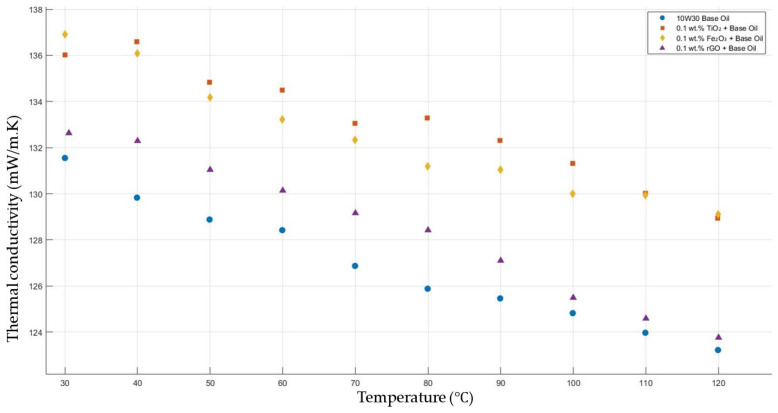
Effect of temperature on thermal conductivity of TiO_2_, Fe_2_O_3_, and rGO 0.1-added nanofluids with base fluid.

Figure 5 compares the thermal conductivity results of 10W30 engine oil with a constant mass fraction of 0.1 of TiO_2_, Fe_2_O_3_, and rGO added within the temperature range 30–120 °C. The graph proves that all nanofluids enhanced the thermal conductivity significantly, while showing the highest enhancements with TiO_2_-, Fe_2_O_3_-, and rGO-added nanofluids, respectively.

### 2.2. Thermal Diffusivity

The rate of heat transfer through a medium is called thermal diffusivity. The higher the thermal diffusivity is, the faster the rate of heat transfer through a medium is.

**Figure 6:**
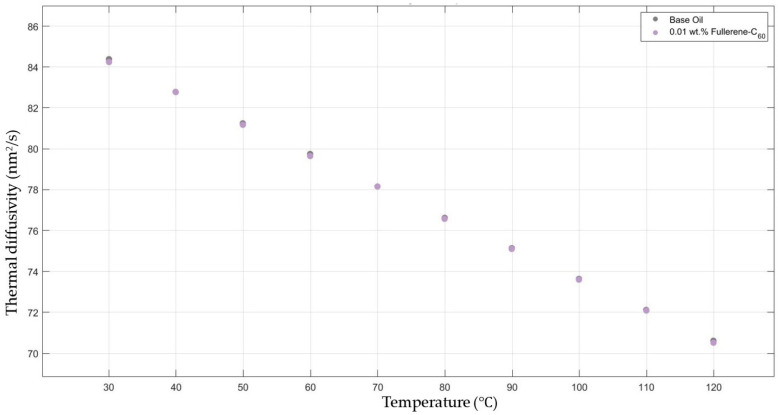
Effect of temperature on thermal diffusivity of fullerene-C_60_ 0.01-added 10W30 engine oil-based nanofluid.

Figure 6 shows the results of the thermal diffusivity values of the base fluid and fullerene-C_60_-added nanofluids with a mass fraction of 0.01 in the temperature range of 30–120 °C.

**Figure 7:**
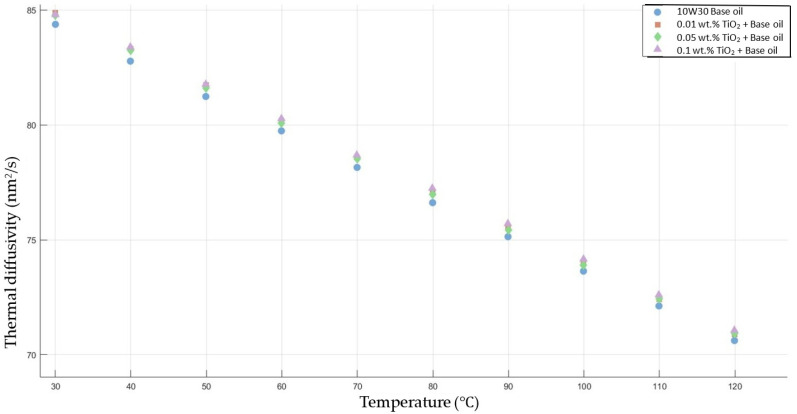
Effect of temperature on thermal diffusivity of TiO_2_ 0.01-, 0.05-, and 0.1-added nanofluids with base fluid.

Figure 7 shows the results of the thermal diffusivity values of the base fluid and TiO_2_-added nanofluids with mass fractions of 0.01, 0.05, and 0.1 in the temperature range of 30–120 °C.

**Figure 8:**
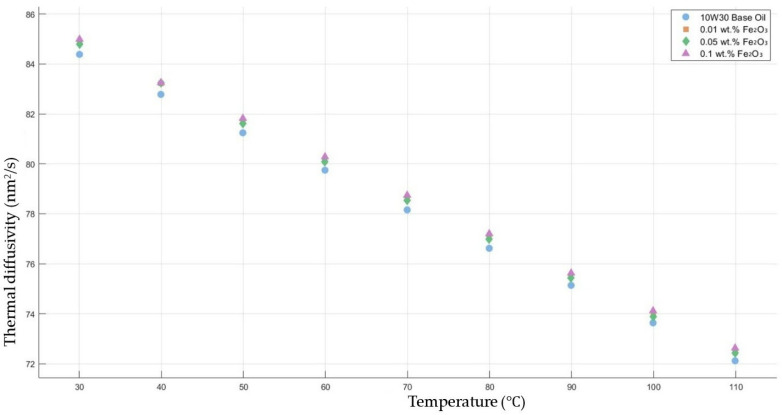
Effect of temperature on thermal diffusivity of Fe_2_O_3_ 0.01-, 0.05-, and 0.1-added nanofluids with base fluid.

Figure 8 shows the results of the thermal diffusivity values of the base fluid and Fe_2_O_3_-added nanofluids with mass fractions of 0.01, 0.05, and 0.1 in the temperature range of 30–120 °C.

**Figure 9:**
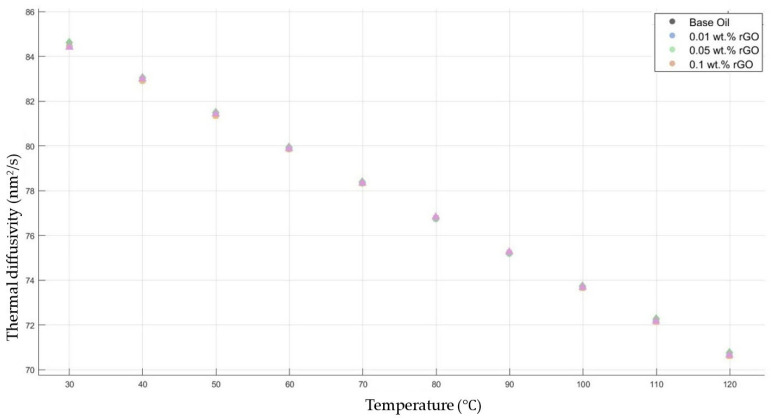
Effect of temperature on thermal diffusivity of rGO 0.01-, 0.05-, and 0.1-added nanofluids with base fluid.

Figure 9 shows the results of the thermal diffusivity values of the base fluid and rGO-added nanofluids with mass fractions of 0.01, 0.05, and 0.1 in the temperature range of 30–120 °C.

**Figure 10:**
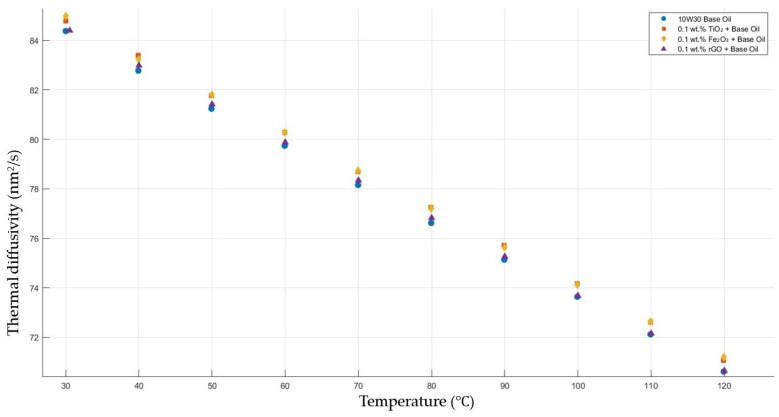
Effect of temperature on thermal diffusivity of TiO_2_, Fe_2_O_3_, and rGO 0.1-added nanofluids with base fluid.

Figure 10 shows the results of the thermal diffusivity values of the base fluid and TiO_2_-, Fe_2_O_3_-, and rGO-added nanofluids with a constant mass fraction of 0.1 in the temperature range of 30–120 °C.

Figure 6, Figure 7, Figure 8, Figure 9 and Figure 10 show the thermal diffusivity vs. temperature graphs with different nanofluids concerning the 10W30 engine oil. As is clearly observed in the figures, there was no significant improvement in the thermal diffusivity of any nanofluid samples with the increment in the temperature compared to that of the base fluid. The thermal diffusivity of the nanofluids and base fluid decreased when increasing the temperature, increasing the gradient of the graphs for both the base fluid and nanofluids within the temperature range of 30–120 °C. These findings tally with the theoretical expectations and are consistent with previous research works [40,41].

### 2.3. Specific Heat Capacity

The quantity of heat absorbed per unit of mass of a material when its temperature increases by one unit can be called the specific heat capacity. A large value of the specific heat capacity of engine oil is most suitable for vehicle engines because it helps to regulate the temperature and increase the cooling rate.

**Figure 11:**
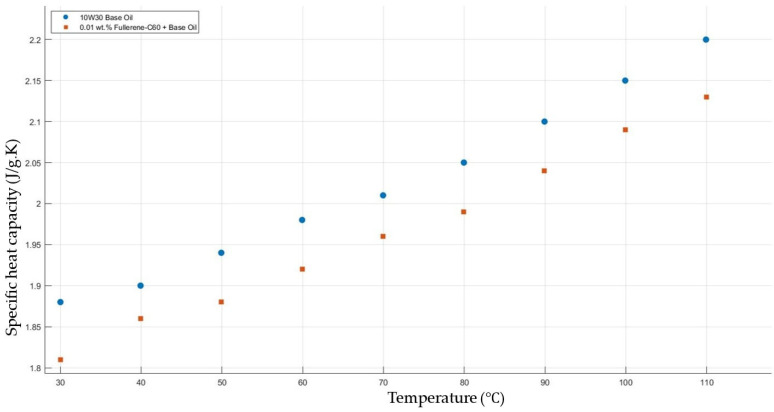
Effect of temperature on the specific heat capacity of fullerene-C_60_ 0.01-added 10W30 engine oil-based nanofluid.

Figure 11 shows the results of specific heat capacity values of the base fluid and fullerene-C_60_-added nanofluids with a mass fraction of 0.01 in the temperature range of 30–120 °C. The experimental results do not show a significant enhancement in the nanofluids’ specific heat capacity in contrast to that of the base fluid.

**Figure 12:**
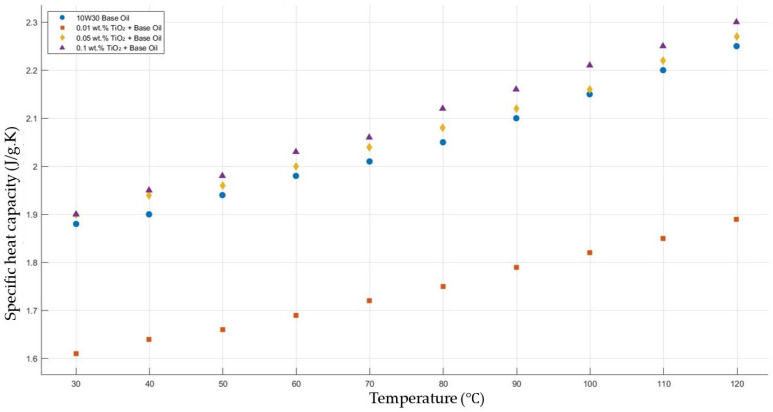
Effect of temperature on the specific heat capacity of TiO_2_ 0.01-, 0.05-, and0.1-added nanofluids with a base fluid.

Figure 12 shows the results of the specific heat capacity values of the base fluid and TiO_2_-added nanofluids with mass fractions of 0.01, 0.05, and 0.1 in the temperature range of 30–120 °C. The results do not show such enhancement in nanofluid-specific heat capacity compared with that of the base fluid. However, the nanofluid with added TiO_2_ with a mass fraction of 0.01 showed a decrease in specific heat capacity compared to the base fluid within the temperature range 30–120 °C.

**Figure 13:**
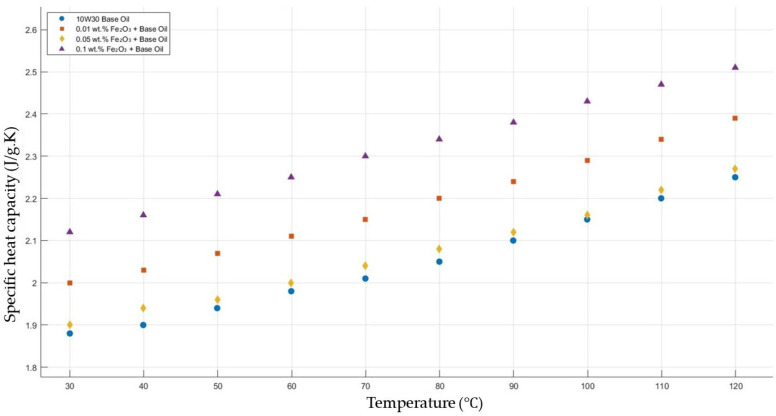
Effect of temperature on the specific heat capacity of Fe_2_O_3_ 0.01-, 0.05-, and 0.1-added nanofluids with the base fluid.

Figure 13 shows the results of the specific heat capacity values of the base fluid and Fe_2_O_3_-added nanofluids with mass fractions of 0.01, 0.05, and 0.1 in the temperature range of 30–120 °C. The experimental results prove that Fe_2_O_3_ with a mass fraction of 0.1 resulted in the highest enhancement in specific heat capacity, while a significant enhancement with Fe_2_O_3_ 0.01 wt.%-added nanofluid was shown, but there was no considerable enhancement in the specific heat capacity of Fe_2_O_3_ 0.05-added nanofluid.

**Figure 14:**
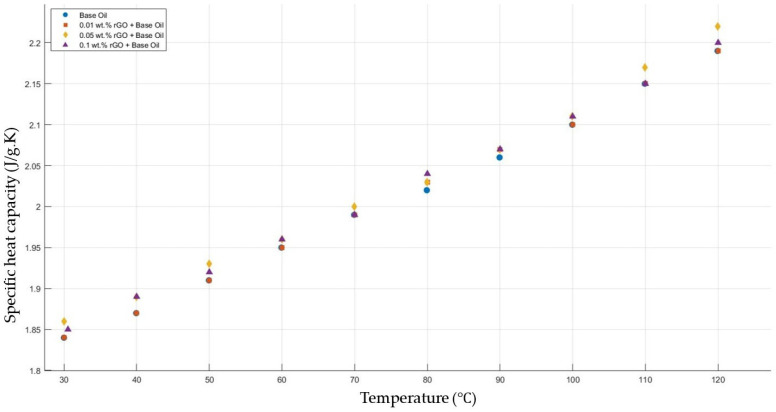
Effect of temperature on the specific heat capacity of rGO 0.01-, 0.05-, and 0.1-added nanofluids with a base fluid.

Figure 14 shows the results of the specific heat capacity values of the base fluid and rGO-added nanofluids with mass fractions of 0.01, 0.05, and 0.1 in the temperature range of 30–120 °C. The results prove that rGO did not affect the specific heat capacity values of the engine oil within the temperature range of 30–120 °C.

**Figure 15:**
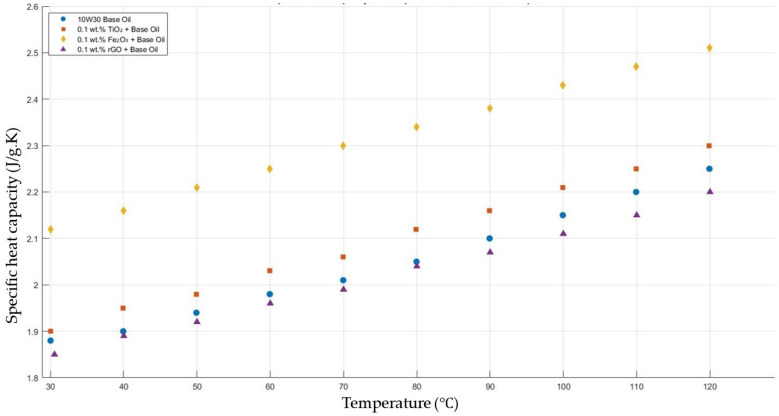
Effect of temperature on the specific heat capacity of TiO_2_, Fe_2_O_3_, and rGO 0.1-added nanofluids with a base fluid.

Figure 15 shows the results of the specific heat capacity values of the base fluid and TiO_2_-, Fe_2_O_3_-, and rGO-added nanofluids with a constant mass fraction of 0.1 in the temperature range of 30–120 °C.

Figure 11, Figure 12, Figure 13, Figure 14 and Figure 15 represent the specific heat capacity vs. temperature graphs of different nanofluids and base fluids. The gradients of the graphs are nearly equal due to increasing the specific heat capacity values when increasing the temperature from 30 to 120 °C. Thermal properties’ enhancements in this study and recent research works are shown in Table 1. However, the thermal properties of the nanofluids used in this study showed enhancement contradicting the expected trend based on the intrinsic thermal conductivity ranking of the nanomaterials. This discrepancy can be initially attributed to differences in particle size, which significantly influence thermal conductivity. Smaller particles tend to increase the effective surface area and improve interaction with the base fluid, while reducing sedimentation rates. Furthermore, agglomeration states and interfacial thermal resistance at the nanoparticle–fluid interface are additional factors that can impact heat transfer behavior. Models other than the Brownian motion, aggregation, and interfacial layering models may also contribute to the observed thermal enhancement. These complex, coupled effects collectively explain the deviations from simple theoretical predictions and highlight the importance of nanoscale interactions in determining thermal performance.

## 3. Materials and Methods

**Figure 16:**
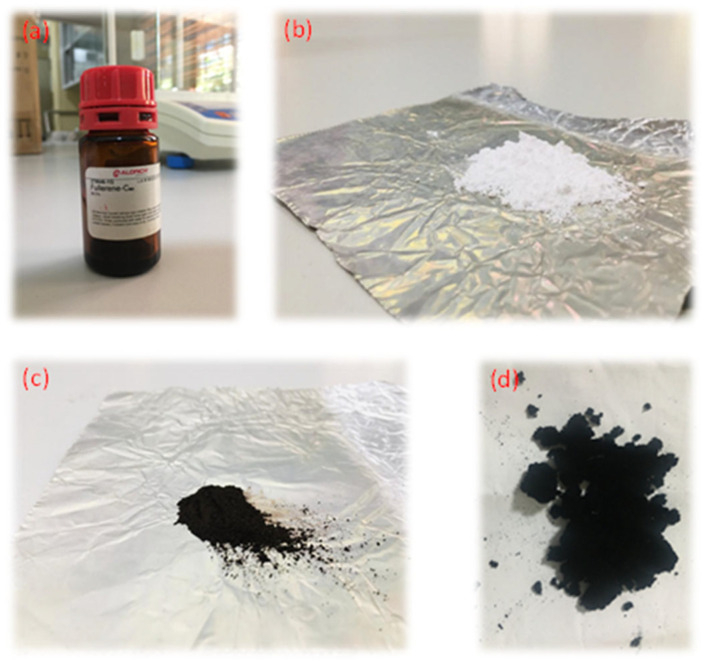
Nanomaterials: (**a**) fullerene-C_60_, (**b**) TiO_2_, (**c**) Fe_2_O_3_, and (**d**) rGO.

In this study, four different nanomaterials were used to prepare nanofluids: fullerene-C_60_ (99.5%, SIGMA-ALDRICH, St. Louis, MO, USA), TiO_2_ (anatase-type, Ningbo Jiweina New Material Technology Co., Ltd., Beijing, China), Fe_2_O_3_ (SLINTEC, Homagama, Sri Lanka), and rGO (Ceylon Graphene Technologies). Figure 16 shows pictures of the four nanomaterials that were directly used for nanofluid preparation.

Three different mass fractions, 0.01, 0.05, and 0.1, were used to prepare nanofluids from each nanoparticle, and their thermal conductivity, thermal diffusivity, and specific heat capacity were measured at the temperature range 30–120 °C with a step size of 10 °C for each sample. The thermal properties of the fullerene-C_60_, TiO_2_, Fe_2_O_3_, and rGO nanoparticles are given in Table 2, whereas the properties of Mobil 10W30 engine oil is given in Table 3.

In this study, Mobil 10W30 engine oil was used as the base fluid for the nanofluids. It is a multi-grade engine oil and is graded according to the standard grading system. Mobil 10W30 engine oil is a very common engine oil in the market and still needs more improvement to enhance the efficiency of engines.

### 3.1. SEM Analysis of Nanoparticles

**Figure 17:**
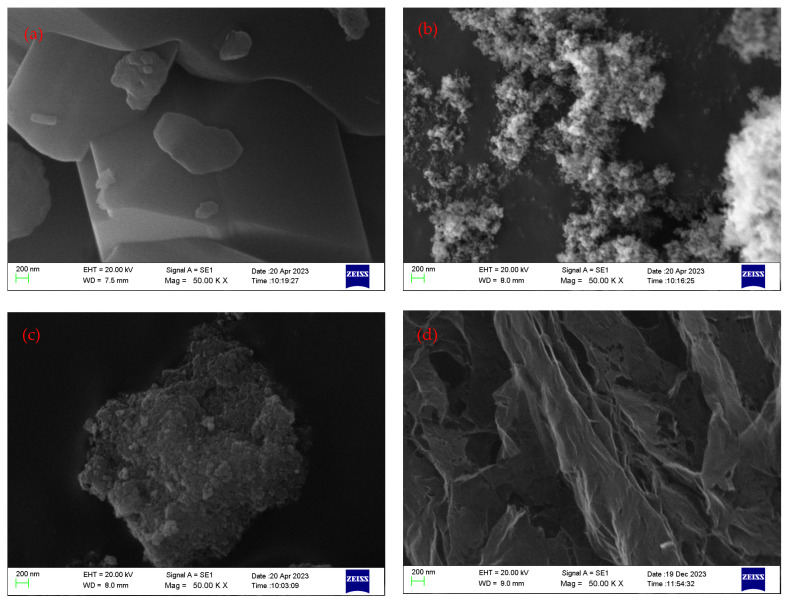
SEM images of the nanoparticles used in this research work. (**a**): fullerene-C_60_; (**b**): TiO_2_; (**c**): Fe_2_O_3_; (**d**): rGO.

The surface topography and composition of the nanoparticles could be investigated using Scanning Electron Microscope (SEM) tests. Figure 17 represents the obtained SEM images of fullerene-C_60_, TiO_2_, Fe_2_O_3_, and rGO, respectively.

### 3.2. Nanofluid Preparation

In this study, Mobile 10W30 engine oil was used as the base fluid to prepare the nanofluids using a two-step method. Equation (1) was used to measure the nanoparticles’ weight to prepare the nanofluids according to the weight ratio. The weights of the base fluid and nanoparticles were measured using the BSA224S-CW (Sartorius AG, Goettingen, Germany; d = 0.1 mg) electronic balance. The measured weights of the five different nanoparticles are presented in Table 4 for different mass fractions of 0.01, 0.05, and 0.1. For thermo-physical measurements, 50 mL of nanofluid was required because 40 ± 2 mL was required for the thermal conductivity meter for thermal measurements.

(1) $$wt.\%=x_{np}/\left(x_{np}+x_{bf}\right)$$

where $x_{np}$ and $x_{bf}$ are the weights of the nanoparticle and base fluid, respectively.

The surface temperature of the base fluid was measured using a thermometer with an accuracy of ±1 °C after the measurements of the weights of the nanoparticles and base fluids. The measured nanoparticles were subsequently added to the measured nanofluids and placed on a magnetic stirrer at 40 °C and 400 rpm for 2 h to disperse the nanoparticles in the engine oil. To obtain better dispersion and eliminate agglomerates, the prepared nanofluid samples were placed in an ultrasonicator (Rocker, SONER 210H, AC, 220 V, 50 Hz, Rocker Scientific Co. Ltd., Kaohsiung City, Taiwan) to expose the samples to ultrasonic waves at 40 °C for 3 h.

**Figure 18:**
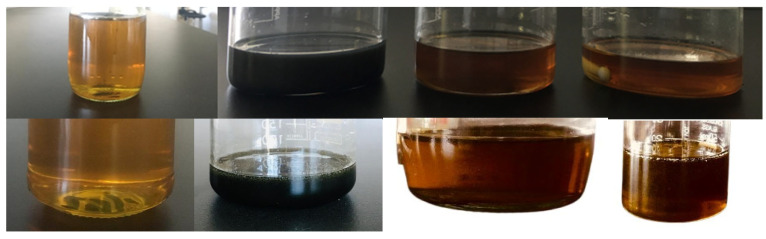
Prepared engine oil-based nanofluid samples before and after two weeks.

This preparation method helped to obtain stable nanofluid samples for at least two weeks without any sedimentation risk; therefore, no surfactant was needed for stabilization. Pictures of prepared nanofluid samples before and after two weeks are shown in Figure 18.

### 3.3. Thermal Property Measurements

**Figure 19:**
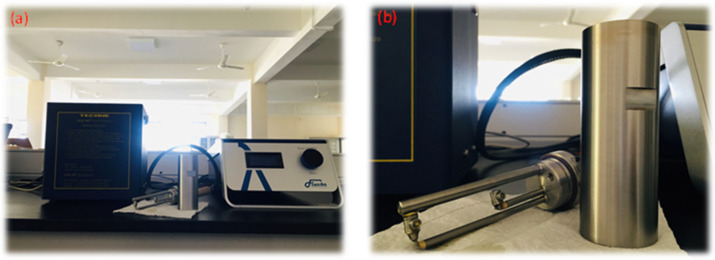
(**a**) LAMBDA thermal conductivity meter and TECHNE UCAL 400+dry block calibrator. (**b**) Sensor and sample container.

The fluids’ thermal properties, such as thermal conductivity, thermal diffusivity, and specific heat capacity, were measured in this experimental work in the temperature range of 30–120 °C with a step size of 10. Three data points were obtained from each sample, and the mean values were analyzed to increase the accuracy of the measurements. There are several methods that can be used to measure the thermal properties of liquids, including the steady state method, temperature oscillation method, transient hot-wire method, etc. [41]. In this experimental work, the transient hot-wire method was used to measure thermal properties using the TECHNE UCAL 400+ dry block calibrator and FLUCON LAMBDA thermal conductivity meter, which could measure the thermal conductivity, thermal diffusivity, and specific heat capacity of both the base fluid and nanofluids, which were measured within the temperature range 30–120 °C. Figure 19 shows the LAMBDA thermal conductivity meter, which uses the transient hot-wire method to measure thermal properties such as thermal conductivity, thermal diffusivity, and specific heat capacity according to the ASTM D7896-19 standardization.

### 3.4. Thermal Property Ratios

The ratios of properties such as thermal conductivity, thermal diffusivity, specific heat capacity, and flash point in the nanofluid and base fluid are discussed in this section.

Equations (2)–(5) can be used to calculate the ratios of thermal conductivity, thermal diffusivity, specific heat capacity, and flash point, respectively.

(2) $$Thermal\;conductivity\;ratio\left(\%\right)=\left(\left(\lambda_{nf}-\lambda_{bf}\right)/\lambda_{bf}\right)\times 100\%$$

where $\lambda_{nf}$ and $\lambda_{bf}$ represent the thermal conductivity of the nanofluid and the thermal conductivity of the base fluid, respectively.

(3) $$Thermal\;diffusivity\;ratio\left(\%\right)=\left(\left(\beta_{nf}-\beta_{bf}\right)/\beta_{bf}\right)\times 100\%$$

where $\beta_{nf}$ and $\beta_{bf}$ represent the thermal diffusivity of the nanofluid and the thermal diffusivity of the base fluid, respectively.

(4) $$Specific\;heat\;capacity\;ratio\left(\%\right)=\left(\left(\gamma_{nf}-\gamma_{bf}\right)/\gamma_{bf}\right)\times 100\%$$

where $\gamma_{nf}$ and $\gamma_{bf}$ represent the specific heat capacity of the nanofluid and the specific heat capacity of the base fluid, respectively.

(5) $$Flash\;point\;ratio\left(\%\right)=\left(\left(\delta_{nf}-\delta_{bf}\right)/\delta_{bf}\right)\times 100\%$$

where $\delta_{nf}$ and $\delta_{bf}$ represent the flash point of the nanofluid and the flash point of the base fluid, respectively.

## 4. Conclusions

Conducting experimental research on engine oil-based nanofluids offers promising avenues for enhancing the performance and efficiency of various engineering systems, particularly in the realm of heat transfer and lubrication. This experimental work contained the preparation procedure of several nanofluids with four different nanoparticles, namely, fullerene-C_60_, TiO_2_, Fe_2_O_3_, and rGO at three mass fractions of 0.01, 0.05, and 0.1, using 10W30 Mobil engine oil as base fluid, and their thermal conductivity, thermal diffusivity, and specific heat capacity were evaluated. The thermal properties were measured within the temperature range of 30–120 °C with a step size of 10 to obtain the best comparative enhancement. The two-step preparation method with stirring at 400 rpm and ultrasonication at 40 °C helped to avoid the sedimentation and agglomeration of nanofluids. The obtained results reveal that most nanoparticles had a significant effect on improving and maintaining the thermal properties of engine oil at standard values under engine operating temperature. The characterization of the nanoparticles showed that the average sizes of fullerene-C_60_, TiO_2_, Fe_2_O_3_, and rGO were 100–200 nm, 30–60 nm, 40–70 nm, and 100–200 nm, respectively, and the prepared nanofluids had good stability, and no precipitation/sedimentation occurred during two weeks. Among the samples, the highest thermal conductivity percentage increment was shown in the TiO_2_/10W30 nanofluid with 0.1 added at 80 °C, and it was 5.8%, while the nanoparticles fullerene-C_60_, TiO_2_, Fe_2_O_3_, and rGO did not significantly affect the thermal diffusivity values of engine oil. Furthermore, the largest specific heat capacity percentage increment was shown in Fe_2_O_3_/10W30 nanofluid with 0.1 added at 70 °C, and it was 14.4%.

It can be concluded that nanoparticles can affect engine oil to increase its thermal properties, while thermal diffusivity is largely unaffected, without compromising its stability at the engine operating temperature to enhance the overall performance of an engine.

## Figures and Tables

**Table 1:** Comparison of thermal property enhancements in recent research works and this study.

Nanomaterial	Base Fluid	Temperature Range	Thermal Conductivity Enhancement	Specific Heat Capacity Enhancement	Ref.
Graphene/TiO_2_	Water	25–75 °C	27.84% at volume fraction 0.5	-	[42]
GO	Water	25–60 °C	19.9% at mass fraction 0.5	-	[43]
Fe_2_O_3_	Water	20–55 °C	90% at volume fraction 3	-	[44]
Fe_2_O_3_	10W30 CALTEX engine oil	80 °C	3.9% at mass fraction 0.01	3.4% at mass fraction 0.01	[36]
TiO_2_	10W30 CALTEX engine oil	40 °C	4.5% at mass fraction 0.01	3.7% at mass fraction 0.01	[36]
TiO_2_	Distilled water	20 °C	10% at volume fraction 1.25	-	[45]
TiO_2_	15W30 engine oil	25–50 °C	20.2% at mass fraction 1	-	[35]
Graphene nanoplates	Water	70 °C	-	28.12% at volume fraction 0.1	[46]
SiO_2_	Base salt	-	-	26.7%	[47]
CNT	Water	60 °C	-	65% at mass fraction 1	[48]
TiO_2_	10W30 Mobil engine oil	80 °C	5.8% at mass fraction 0.1	-	This study
Fe_2_O_3_	10W30 Mobil engine oil	70 °C	-	14.4% at mass fraction 0.1	This study

**Table 2:** Thermo-physical properties of fullerene-C_60_, TiO_2_, Fe_2_O_3_, and rGO nanoparticles.

Property	Mobil 10W30
Density (Kg/m^3^) at 30 °C	830.74
Thermal conductivity (W/mK) at 30 °C	0.13152
Thermal conductivity (W/mK) 100 °C	0.12482
Thermal diffusivity (nm^2^/S) at 30 °C	84.383
Thermal diffusivity (nm^2^/S) at 100 °C	73.637

**Table 3:** Thermo-physical properties of Mobil 10W30 engine oil.

Property	Fullerene-C_60_	TiO_2_	Fe_2_O_3_	rGO
Density (g/cm^3^)	1.65	3.78	5.24	0.0059
Size (nm)	100–200	20–40	30–80	100–200
Thermal conductivity (W/mK)	0.2	4	0.58	46.1
Purity (%)	99.5	99	98	98
Appearance	Black powder	White powder	Red–brown powder	Soft black powder platelets

**Table 4:** Weights of nanomaterials for three different mass fractions.

Weight Fraction (wt.%)	Fullerene-C_60_ (g)	TiO_2_ (g)	Fe_2_O_3_ (g)	rGO (g)
0.01	0.0040	0.0071	0.0076	0.0053
0.05	-	0.0281	0.0274	0.0289
0.1	-	0.0526	0.0524	0.0529

## Data Availability

Data are contained within this article.
